# Silencing lncRNA AGAP2-AS1 Upregulates miR-195-5p to Repress Migration and Invasion of EC Cells via the Decrease of FOSL1 Expression

**DOI:** 10.1016/j.omtn.2019.12.036

**Published:** 2020-01-15

**Authors:** Sining Shen, Ke Li, Ying Liu, Xianben Liu, Baoxing Liu, Yufeng Ba, Wenqun Xing

**Affiliations:** 1 Department of Thoracic Surgery, Affiliated Cancer Hospital of Zhengzhou University (Henan Cancer Hospital), Zhengzhou 450008, P.R. China; 2 Department of Oncology, Affiliated Cancer Hospital of Zhengzhou University (Henan Cancer Hospital), Zhengzhou 450008, P.R. China

**Keywords:** esophageal cancer, long non-coding AGAP2-AS1, microRNA-195-5p, FOSL1, proliferation, migration, invasion, apoptosis

## Abstract

The interaction of long non-coding RNAs (lncRNAs), microRNAs (miRNAs), and mRNAs has been implicated in various types of cancers, including esophageal cancer (EC). The current study aimed to investigate the role of AGAP2-AS1/miR-195-5p/Fos-like antigen-1 (FOSL1) in EC progression. The expression of AGAP2-AS1, miR-195-5p, and FOSL1 in tumor tissues isolated from EC patients and EC cell lines was determined by quantitative reverse transcriptase polymerase chain reaction (qRT-PCR), the results of which illustrated that AGAP2-AS1 and FOSL1 were increased while miR-195-5p was reduced in EC. Next, the ectopic expression, knockdown, and reporter assay experiments were all employed to elucidate the mechanism of AGAP2-AS1/miR-195-5p/FOSL1 in the processes of EC cell proliferation, cell cycle, apoptosis, invasion, and migration as well as tumor growth. Knockdown of AGAP2-AS1 or overexpression of miR-195-5p reduced EC cell proliferation, migration, and invasion, blocked cell cycle entry, and elevated apoptosis. FOSL1 was found to be specifically targeted by miR-195-5p. AGAP2-AS1 was observed to upregulate FOSL1 by binding to miR-195-5p. Silencing of AGAP2-AS1 was observed to restrain the development of EC both *in vitro* and *in vivo* through upregulating miR-195-5p and downregulating FOSL1. Taken together, AGAP2-AS1 knockdown exercises suppressive effects on the development of EC through miR-195-5p-dependent downregulation of FOSL1. Therefore, targeting AGAP2-AS1 could be a future direction to develop a novel molecule-targeted therapeutic strategy for EC.

## Graphical Abstract

**Figure:**
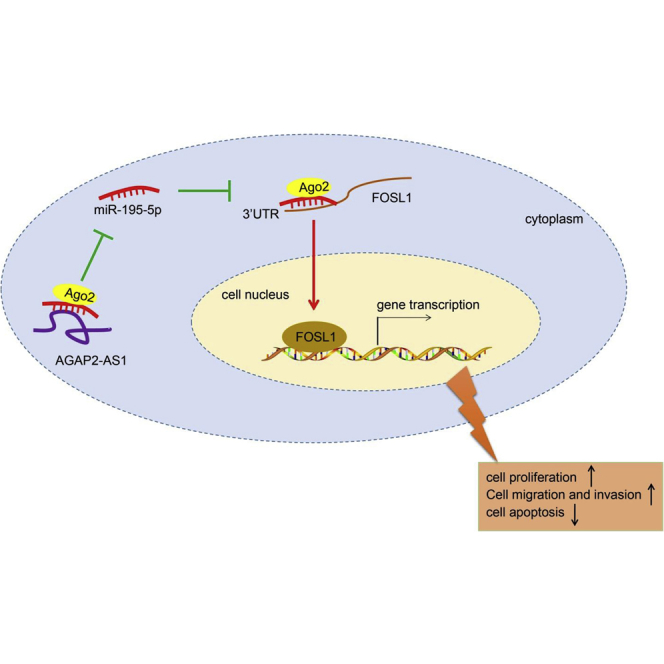

## Introduction

As one of the most deadly diseases worldwide, esophageal cancer (EC) is a malignancy characterized by rapid progression and poor patient prognosis.^1^ The risk factors associated with EC include cigarette smoking, alcohol consumption, hot tea drinking, red meat consumption, bad oral hygiene, low fruit and vegetable intake, as well as low socioeconomic conditions.^2^ Although tremendous strides have been made in the treatment of EC, such as chemotherapy and surgical resection, patients diagnosed with EC still have poor prognosis, with a relatively low 5-year survival of only about 10%.^3^^,^^4^ Long non-coding RNAs (lncRNAs), longer than 200 nt, have been highlighted to be essential for the tumorigenesis and progression of EC.^5^^,^^6^ High expression of AGAP2-AS1 has been identified in gastric cancer and non-small-cell lung cancer, suggesting that knockdown of AGAP2-AS1 leads to a decrease in cell proliferation and migration, along with the repression of invasion and tumorigenesis.^7^^,^^8^ However, it remains unknown as to whether AGAP2-AS1 influences cancer progression in EC.

Additionally, microRNAs (miRNAs), endogenous non-protein-coding small RNA molecules, have been implicated in a wide array of physiological processes, including cell proliferation, invasion, apoptosis, and self-renewal.^9^ A previous study revealed downregulation of miR-195 in esophageal squamous cell carcinoma (ESCC), which indicates that the ectopic expression of miR-195 diminishes ESCC cell proliferation and invasion.^10^ Previous evidence indicates that lncRNAs can function as miRNA sponges by directly interacting with miRNAs.^11^^,^^12^ Fos-like antigen-1 (FOSL1), belonging to the activator protein-1 transcription factor, has been suggested to be a potential biomarker of various human cancers.^13^ The association between increased FOSL1 expression and ESCC has been investigated, and the silencing of FOSL1 has been reported to have inhibitory effects on cell proliferation and invasion and confer protection against the cytotoxicity of cisplatin in ESCC cells.^14^^,^^15^ It was presumed that there were putative binding sites between miR-195-5p and FOSL1 based on the miRNA-mRNA interaction analysis from the StarBase website, as well as putative binding sites between miR-195-5p and AGAP2-AS1 through lncRNA-miRNA interaction prediction by the StarBase and RAID databases. As discussed above, we hypothesized that AGAP2-AS1 plays a critical role in EC development with the implication of miR-195-5p and FOSL1. Hence, the central objective of the current study was to investigate the relationships among AGAP2-AS1, miR-195-5p, and FOSL1, and their regulatory mechanism influencing cell proliferation, invasion, migration, and apoptosis.

## Results

### miR-195-5p Is Poorly Expressed in EC Tissues and Cells

**Figure 1:**
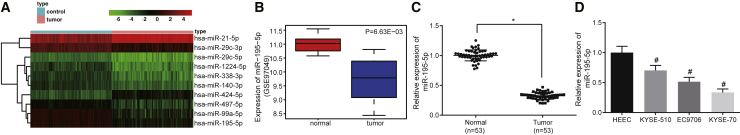
Reduced Expression of miR-195-5p Is Identified in EC Tissues and Cell Lines (A) Heatmap of 10 differentially expressed miRNAs in the GEO: GSE43732 expression dataset. The y axis represents the differentially expressed miRNAs. The upper right bar refers to color gradation, and each box indicates the expression level of a gene in a sample. (B) Expression of miR-195-5p in EC and normal samples obtained from the GEO: GSE97049 expression dataset. (C) Relative expression of miR-195-5p in EC tumor tissues and their corresponding adjacent normal tissues (n = 53) determined by qRT-PCR. (D) Expression of miR-195-5p in EC cell lines (KYSE70, KYSE-510, EC9706) and HEECs determined by qRT-PCR. *p < 0.05 compared with normal tissues; ^#^p < 0.05 compared with HEECs. Data in (B) and (C) are expressed as mean ± standard deviation and compared by a paired t test. Data in (D) are expressed as mean ± standard deviation, compared by a one-way ANOVA, followed by Tukey’s *post hoc* test. The experiment was repeated three times.

Initially, the differential analysis of EC expression datasets was performed using R language. The expression heatmap of the top 10 differentially expressed miRNAs screened from GEO: GSE43732 is depicted in Figure 1A, among which two miRNAs were found to be upregulated, and the others were downregulated in EC. Specifically, miR-195-5p was also identified to be poorly expressed in EC based on another dataset, GEO: GSE97049 (Figure 1B). The existing literature has suggested that miR-195-5p plays an anti-oncogenic role in a variety of cancers, including non-small-cell lung cancer,^16^ colorectal cancer,^17^ and gastric cancer.^18^ However, the regulatory role of miR-195-5p in EC remains unknown.

To verify the role of miR-195-5p in EC, the expression of miR-195-5p was characterized in EC tissue samples and their corresponding adjacent normal tissues from 53 EC patients. miR-195-5p was expressed at a lower level in EC tissues when compared to the adjacent normal tissues (p < 0.05; Figure 1C). At the same time, it was consistently demonstrated that when compared with normal human immortalized esophageal epithelial cells (HEECs), miR-195-5p was poorly expressed in EC cell lines (KYSE70, KYSE-510, and EC9706), with KYSE70 cells showing the lowest expression (Figure 1D). Hence, KYSE70 cells were selected for subsequent experiments. Based on the aforementioned results, miR-195-5p is under-expressed in EC tissues and cells.

### Overexpressing miR-195-5p Inhibits Proliferation and Migration and Induces Cell Cycle Arrest and Apoptosis of EC Cells

**Figure 2:**
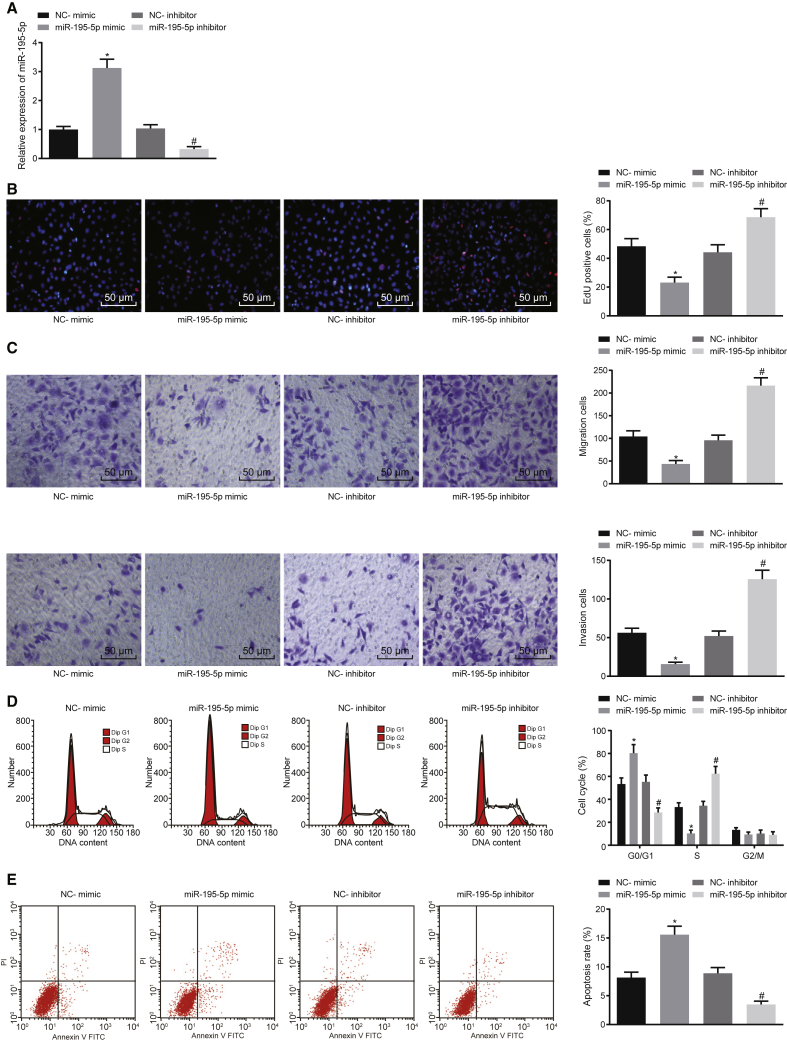
miR-195-5p Inhibits Proliferation and Migration and Promotes Apoptosis of EC Cells KYSE70 cells were transfected with miR-195-5p mimic or miR-195-5p inhibitor with NC-mimic or NC-inhibitor as controls. (A) Relative expression of miR-195-5p in the transfected EC cells determined by qRT-PCR. (B) Proliferation of the transfected EC cells assessed by EdU assay (original magnification, ×200). (C) Migration and invasion of the transfected EC cells evaluated by Transwell assay (original magnification, ×200). (D) Cell cycle analysis of the transfected EC cells evaluated by flow cytometric PI single staining. (E) Apoptosis of the transfected EC cells assessed by flow cytometric annexin V-FITC/PI double staining. *p < 0.05 compared with KYSE70 cells transfected with NC-mimic; ^#^p < 0.05 compared with KYSE70 cells transfected with NC-inhibitor. The measurement data are expressed as mean ± standard deviation. Data between two groups were compared by an unpaired Student’s t test, while data among multiple groups were compared by one-way ANOVA, followed by Tukey’s *post hoc* test. The experiment was repeated three times.

Next, in order to evaluate the detailed effects associated with miR-195-5p on EC cells, the expression of miR-195-5p was altered in KYSE70 cells. The transfection efficiency was then determined in the KYSE70 cells, which revealed that transfection of the miR-195-5p mimic increased miR-195-5p expression, while transfection with the miR-195-5p inhibitor reduced miR-195-5p expression (Figure 2A). The proliferation (Figure 2B), migration, and invasion (Figure 2C) along with cell cycle and apoptosis (Figures 2D and 2E) of the KYSE70 cells were also examined in response to transfection with miR-195-5p mimic or inhibitor. When miR-195-5p expression was restored in the KYSE70 cells, cell proliferation, migration, and invasion were reduced. In contrast, inhibition of miR-195-5p elevated KYSE70 cell proliferation, migration, and invasion. Following overexpression of miR-195-5p, the proportion of KYSE70 cells at the G_0_/G_1_ phase was elevated while the proportion at the S phase was reduced, suggesting an inhibited cell cycle progression, while an opposite trend was observed whereby the cell cycle progression was enhanced after inhibition of miR-195-5p. Additionally, upregulation of miR-195-5p was found to notably enhance KYSE70 cell apoptosis, while the miR-195-5p inhibitor decreased KYSE70 cell apoptosis. Taken together, these results indicate that miR-195-5p inhibits the proliferation and migration of EC cells, while enhancing the apoptosis of EC cells.

### FOSL1, Highly Expressed in EC, Is a Target Gene of miR-195-5p

**Figure 3:**
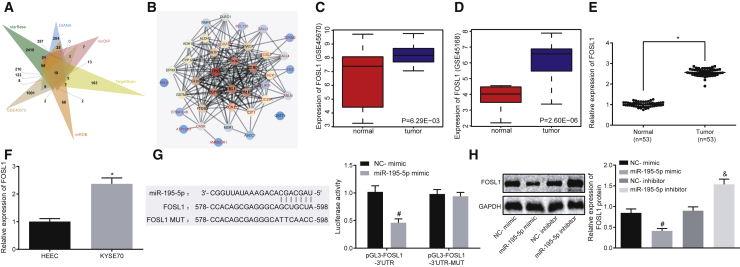
As a Target Gene of miR-195-5p, FOSL1 Is Highly Expressed in EC (A) Intersection of the target genes of miR-195-5p predicted by StarBase, DIANA, mirDIP, TargetScan, and miRDB and DEGs in the GEO: GSE45670 dataset. (B) PPI network of top 20 EC-related genes from DisGeNET and the 18 intersected DEGs from panel, in which red characters indicate DEGs that bind to miR-195-5p, black characters indicate EC-related genes, and circle color indicates the correlation degree with other genes where red indicates close correlation. (C) Relative expression of FOSL1 in EC and normal samples based on the GEO: GSE45670 dataset. (D) Relative expression of FOSL1 in EC and normal samples based on the GEO: GSE45168 dataset. (E) Expression of FOSL1 in EC tissues and adjacent normal tissues (n = 53) determined by qRT-PCR. (F) Expression of FOSL1 in EC cell line KYSE70 and HEECs determined by qRT-PCR. (G) Relationship between miR-195-5p and FOSL1 analyzed by detecting the luciferase activity of pGL3-FOSL1-3′ UTR and pGL3-FOSL1-3′ UTR-MUT after transfection with miR-195-5p mimic or NC-mimic through dual-luciferase reporter assay. (H) Expression of FOSL1 in KYSE70 cells transfected with miR-195-5p mimic or miR-195-5p inhibitor measured by western blot analysis. *p < 0.05 compared with adjacent normal tissues or HEECs; ^#^p < 0.05 compared with KYSE70 cells transfected with NC-mimic; ^&^p < 0.05 compared with KYSE70 cells transfected with NC-inhibitor. The measurement data are expressed as mean ± standard deviation. Data in (C)–(E) were compared by paired t test. Data in (F)–(H) were compared by unpaired Student’s t test. Data among multiple groups were compared by one-way ANOVA and Tukey’s *post hoc* test. The experiment was repeated three times.

The target genes of miR-195-5p were predicted based on data from the StarBase, DIANA, mirDIP, TargetScan, and miRDB databases. 870 target genes from mirDIP (integrated score > 0.7), 1,094 target genes from miRDB (target score > 60), 4,347 target genes from StarBase, 1,595 target genes from DIANA, and 1,504 target genes from TargetScan were predicted in accordance with the aforementioned databases. The target genes were intersected with EC-related genes in GEO: GSE45670, which identified 18 intersection genes (FOSL1, CHEK1, E2F7, BCL11B, SALL1, CCNE1, SALL4, CDC25A, ATP13A3, TENM2, HOXA10, ANLN, EN2, PLAG1, EPB41L4B, CASK, AMMECR1, APLN) that were overexpressed in EC and could potentially be regulated by miR-195-5p (Figure 3A). The top 20 EC-related genes (TGFBR2, MSR1, RNF6, ASCC1, DLEC1, WWOX, TP53, ALDH2, GSTM1, EGFR, CYP1A1, ADH1B, CCND1, ERBB2, GSTP1, GSTT1, EPHX1, PTGS2, CDKN2A, EGF) identified from DisGeNET and the 18 differentially expressed genes (DEGs) binding to miR-195-5p were selected, with the protein-protein interaction (PPI) network of those genes mapped using the String database (Figure 3B). CCNE1 and FOSL1 were located in the core of PPI network, highlighting that they possess the strongest correlations with the other DEGs, and possibly have a significant potential in relationship to EC progression.

FOSL1 was upregulated in EC according to GEO: GSE45670 (Figure 3C) and GEO: GSE45168 (Figure 3D), which was further verified by determination of FOSL1 expression in EC tissues and cells (Figures 3E and 3F). Based on the miR-195-5p binding sites on FOSL1 predicted by StarBase, we mutated the binding sites for miR-195-5p on FOSL1 3′ untranslated region (UTR) and constructed wild-type (pGL3-FOSL1-3′ UTR) and mutant (pGL3-FOSL1-3′ UTR-MUT) luciferase reporter gene plasmids. The luciferase activity of pGL3-FOSL1-3′ UTR was significantly decreased in the cells co-transfected with miR-195-5p mimic (p < 0.05), while the luciferase activity of pGL3-FOSL1-3′ UTR-MUT exhibited no significant change between co-transfection with miR-195-5p mimic and negative control (NC)-mimic (p > 0.05; Figure 3G), indicating that FOSL1 was indeed a target gene of miR-195-5p. Next, the expression of FOSL1 was determined in the KYSE70 cells transfected with miR-195-5p mimic or miR-195-5p inhibitor. The results illustrated that the expression of FOSL1 was significantly decreased in the EC cells transfected with miR-195-5p mimic, but increased in EC cells transfected with miR-195-5p inhibitor (Figure 3H). Hence, we concluded that FOSL1 was a target gene of miR-195-5p and was an upregulated gene in EC.

### miR-195-5p Represses Proliferation, Migration, and Invasion and Induces Apoptosis of EC Cells by Targeting FOSL1

**Figure 4:**
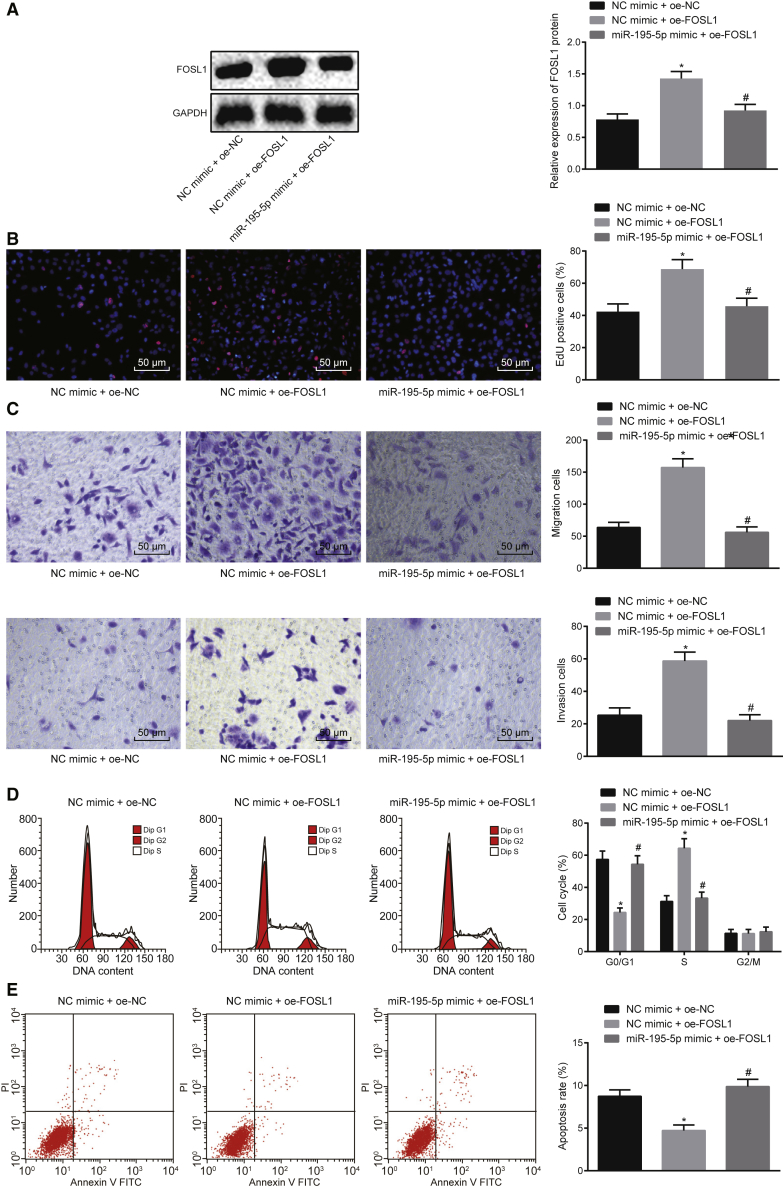
Ectopic Expression of miR-195-5p Contributes to Repressed Proliferation, Migration, and Invasion, Yet Potentiated Apoptosis of EC Cells through Targeting FOSL1 KYSE70 cells were co-transfected with oe-FOSL1 in the presence of miR-195-5p mimic or NC-mimic. (A) Protein expression of FOSL1 in the transfected KYSE70 cells measured by western blot analysis. (B) Proliferation of the transfected KYSE70 cells detected by EdU assay (original magnification, ×200). (C) Migration and invasion of the transfected KYSE70 cells evaluated by Transwell assay (original magnification, ×200). (D) Proportion of the transfected KYSE70 cells at G_0_/G_1_ and S phases assessed by flow cytometric PI single staining. (E) Apoptosis of the transfected KYSE70 cells assessed by flow cytometry. *p < 0.05 compared with KYSE70 cells treated with NC-mimic + oe-NC; ^#^p < 0.05 compared with KYSE70 cells treated with NC-mimic + oe-FOSL1. The measurement data are expressed as mean ± standard deviation and compared by one-way ANOVA, followed by Tukey’s *post hoc* test. The experiment was repeated three times.

In order to elucidate the effect of miR-195-5p targeting FOSL1 on the biological characteristics of EC cells *in vitro*, KYSE70 cells were co-transfected with NC-mimic and overexpressed (oe)-NC, NC-mimic and oe-FOSL1, or miR-195-5p mimic and oe-FOSL1. Next, the expression of FOSL1 was evaluated in the transfected KYSE70 cells, which revealed that the expression of FOSL1 was significantly increased in the EC cells co-transfected with NC-mimic and oe-FOSL1, but oe-FOSL1-induced expression of FOSL1 was decreased in the cells co-transfected with miR-195-5p mimic (Figure 4A). Meanwhile, the EC cell proliferation (Figure 4B), migration, invasion (Figure 4C), cell cycle (Figure 4D), and apoptosis (Figure 4E) were measured following co-transfection. The results indicated that the proliferation, migration, and invasion of EC cells were significantly enhanced following the overexpression of FOSL1 (p < 0.05). The proliferation, migration, and invasion of EC cells co-transfected with miR-195-5p mimic and oe-FOSL1 were decreased when compared with those co-transfected with NC-mimic and oe-FOSL1 (p < 0.05). The proportion of cells at the G_0_/G_1_ phase was significantly decreased, but that at the S phase was increased following overexpression of FOSL1 (p < 0.05). The proportion of G_0_/G_1_ phase cells co-transfected with miR-195-5p mimic and oe-FOSL1 was significantly increased but that at the S phase was reduced when compared with that of cells co-transfected with NC-mimic and oe-FOSL1 (p < 0.05). The apoptosis ability of the EC cells was significantly lowered following the overexpression of FOSL1 (p < 0.05). The apoptosis ability of EC cells co-transfected with miR-195-5p mimic and oe-FOSL1 was higher than that for EC cells co-transfected with NC-mimic and oe-FOSL1 (p < 0.05). Therefore, miR-195-5p inhibited proliferation, migration, and invasion and promoted apoptosis of EC cells through targeting FOSL1.

### AGAP2-AS1 Binds to miR-195-5p to Upregulate the Expression of FOSL1

**Figure 5:**
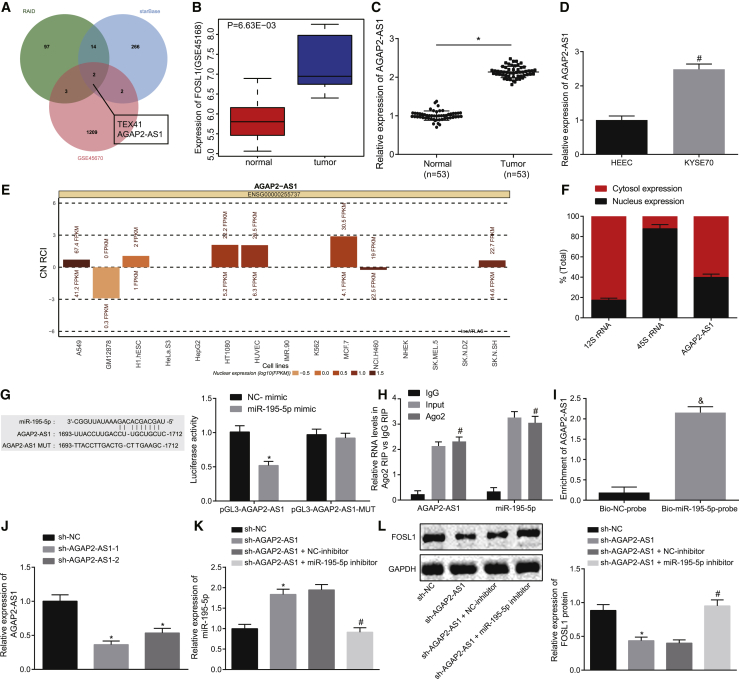
AGAP2-AS1 Upregulates FOSL1 Expression by Binding to miR-195-5p (A) Intersection of potential lncRNAs binding to miR-195-5p predicated using RAID and StarBase and differentially expressed lncRNAs analyzed from the GEO: GSE45670 dataset. (B) Expression of AGAP2-AS1 in EC and normal samples based on the GEO: GSE45670 dataset. *p < 0.05 compared with normal samples. (C) Expression of AGAP2-AS1 in EC tissues and adjacent normal tissues (n = 53) determined by qRT-PCR. *p < 0.05 compared with adjacent normal tissues. (D) Expression of AGAP2-AS1 in KYSE70 and HEECs determined by qRT-PCR. ^#^p < 0.05 compared with HEECs. (E) Subcellular localization of AGAP2-AS1 analyzed based on the LncATLAS database, where CN RCI (relative concentration index) indicates the ratio of lncRNA expression in the cytoplasm to that in the nucleus of the cell line. FPKM, fragments per kilobase of exon model per million mapped fragments. (F) Nuclear and cytoplasmic expression of AGAP2-AS1 in EC cell line KYSE70 determined by qRT-PCR. (G) Relationship between AGAP2-AS1 and miR-195-5p identified by detecting luciferase activity of pGL3-AGAP2-AS1 and pGL3-AGAP2-AS1-MUT after transfection with miR-195-5p mimic or NC-mimic through dual-luciferase reporter assay. *p < 0.05 compared with cells transfected with NC-mimic. (H) Relative levels of AGAP2-AS1 and miR-195-5p precipitated by Ago2 detected by RIP. ^#^p < 0.05 compared with IgG RIP. (I) Enrichment of AGAP2-AS1 by Bio-miR-195-5p-probe and Bio-NC-probe detected by RNA pull-down assay. ^&^p < 0.05 compared with Bio-NC-probe. (J) Relative expression of AGAP2-AS1 in EC cells transfected with short hairpin RNA (sh)-AGAP2-AS1-1 and sh-AGAP2-AS1-2 determined by qRT-PCR. (K) Relative expression of miR-195-5p in EC cells after overexpressing or silencing AGAP2-AS1 determined by qRT-PCR. (L) Protein expression of FOSL1 in EC cells after overexpressing or silencing AGAP2-AS1 measured by western blot analysis. In (J)–(L), *p < 0.05 compared with cells transfected with sh-NC; ^#^p < 0.05 compared with cells co-transfected with sh-AGAP2-AS1 and NC-inhibitor. The measurement data are expressed as mean ± standard deviation. Data in (B) and (C) were compared by paired t test. Data in (D), (G), and (I) were compared by unpaired Student’s t test. Data in (H) and (J)–(L) were compared by one-way ANOVA and Tukey’s *post hoc* test. The experiment was repeated three times.

lncRNAs that possess the potential of binding to miR-195-5p were predicted based on the databases RAID and StarBase. After comparing the predicted potential lncRNAs with the upregulated lncRNAs identified from GEO: GSE45670, two lncRNAs (TEX41 and AGAP2-AS1) were found in the intersection (Figure 5A). It has been reported that AGAP2-AS1 can promote the development of gastric cancer^7^ and breast cancer,^19^ while the effect of AGAP2-AS1 on EC development remains unclear. AGAP2-AS1 was identified to be upregulated in EC through analyzing the lncRNA expression dataset GEO: GSE45670 (Figure 5B), which was further verified in EC tissue samples and cells (Figures 5C and 5D). In the LncATLAS database, AGAP2-AS1 was observed to be localized in the cytoplasm in multiple cell lines (Figure 5E). Nuclear/cytoplasmic RNAs were separated, and the nuclear and cytoplasmic expression of AGAP2-AS1 was determined by quantitative reverse transcriptase polymerase chain reaction (qRT-PCR) in the EC cell line KYSE70, which demonstrated that AGAP2-AS1 was expressed in both the nucleus and cytoplasm of KYSE70 cells (Figure 5F). Therefore, AGAP2-AS1 was speculated to bind to miR-195-5p to regulate FOSL1.

In order to validate our hypothesis, a dual-luciferase reporter assay was initially performed. As shown in Figure 5G, the luciferase activity of pGL3-AGAP2-AS1 was obviously decreased in the cells co-transfected with miR-195-5p mimic when compared with the cells co-transfected with NC-mimic (p < 0.05), while the luciferase activity of pGL3-AGAP2-AS1-MUT exhibited no significant change (p > 0.05). An RNA immunoprecipitation (RIP) assay was then performed, which illustrated that the levels of AGAP2-AS1 and miR-195-5p were significantly higher when precipitated with Ago2 than with immunoglobulin G (IgG) (p < 0.05; Figure 5H). An RNA pull-down assay also revealed that the enrichment of AGAP2-AS1 was higher in cells treated with biotin-labeled (Bio)-miR-195-5p-probe than in cells treated with Bio-NC-probe (p < 0.05; Figure 5I). To further investigate the mechanism by which AGAP2-AS1 regulates miR-195-5p and FOSL1, the expression of AGAP2-AS1 was knocked down by short hairpin RNAs (shRNAs) in EC cells. As shown in Figure 5J, the knockdown efficiency of short hairpin RNA (sh)-AGAP2-AS1-1 was found to be the highest. Hence, sh-AGAP2-AS1-1 was used for subsequent knockdown experiments. The KYSE70 cells were transfected with sh-NC, sh-AGAP2-AS1, or co-transfected with sh-AGAP2-AS1 and NC-inhibitor or sh-AGAP2-AS1 and miR-195-5p inhibitor to analyze the effects of AGAP2-AS1 on expression of miR-195-5p and FOSL1. The expression of miR-195-5p was significantly increased, while FOSL1 expression was significantly decreased in the EC cells following the silencing of AGAP2-AS1, which was reversed by co-transfection with miR-195-5p mimic (Figures 5K and 5L). Taken together, the aforementioned data demonstrated that AGAP2-AS1 promoted the expression of FOSL1 by sponging miR-195-5p.

### AGAP2-AS1 Downregulates miR-195-5p and Upregulates FOSL1 to Regulate the Proliferation, Migration, Invasion, and Apoptosis of EC Cells

**Figure 6:**
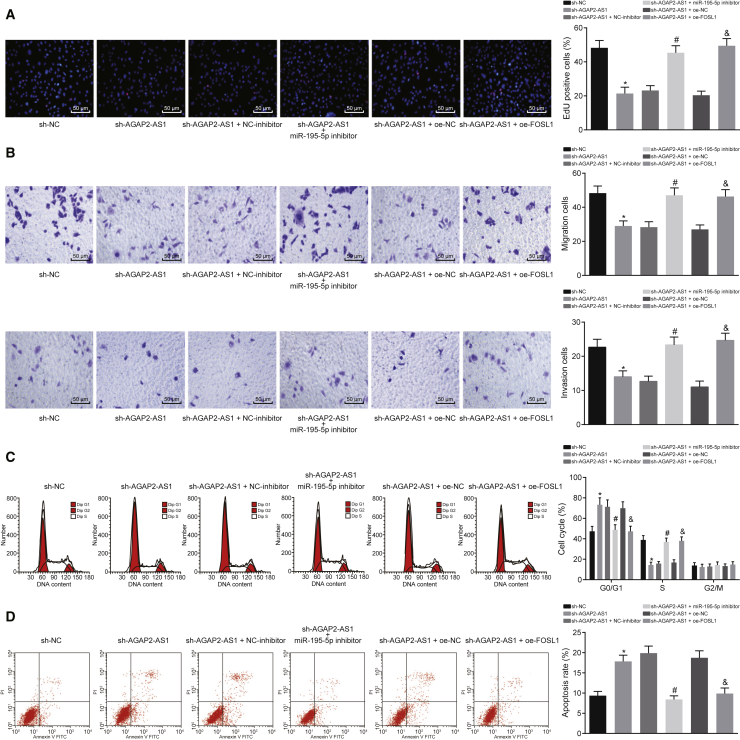
AGAP2-AS1 Silencing Inhibits Proliferation, Migration, and Invasion but Promotes Apoptosis of EC Cells by Decreasing FOSL1 Expression via Upregulation of miR-195-5p KYSE70 cells were transfected with sh-AGAP2-AS1 alone or co-transfected with sh-AGAP2-AS1 and miR-195-5p inhibitor or oe-FOSL1. (A) Proliferation of the transfected KYSE70 cells assessed by EdU assay (original magnification, ×200). (B) Migration and invasion of KYSE70 cells evaluated by Transwell assay (original magnification, ×200). (C) Cell cycle distribution of EC cells assayed by flow cytometric PI single staining. (D) Apoptosis of EC cells assayed by flow cytometric annexin V-FITC/PI double staining. *p < 0.05 compared with KYSE70 cells transfected with sh-NC; ^#^p < 0.05 compared with KYSE70 cells co-transfected with sh-AGAP2-AS1 and NC-inhibitor; ^&^p < 0.05 compared with KYSE70 cells co-transfected with sh-AGAP2-AS1 and oe-NC. The measurement data are expressed as mean ± standard deviation, and compared by one-way ANOVA and Tukey’s *post hoc* test. The experiment was repeated three times.

To explore the mechanism by which AGAP2-AS1 regulates EC cell activities, AGAP2-AS1 and miR-195-5p were downregulated and FOSL1 was upregulated in the KYSE70 cells. Next, the proliferation (Figure 6A), migration, invasion (Figure 6B), cell cycle (Figure 6C), and apoptosis (Figure 6D) of KYSE70 cells were evaluated. After AGAP2-AS1 was silenced, the proliferation, migration, and invasion of KYSE70 cells were significantly decreased, which could be rescued by inhibition of miR-195-5p or overexpression of FOSL1. The proportion of cells at the G_0_/G_1_ phase was significantly elevated, while the proportion of cells at the S phase was decreased along with promoted apoptosis after sh-AGAP2-AS1 transfection, all of which was reversed by the co-transfection with either miR-195-5p inhibitor or oe-FOSL1. Thus, AGAP2-AS1 functioned as an inhibitor of miR-195-5p to upregulate FOSL1 and consequently promote proliferation, migration, and invasion, but to block cell cycle arrest and apoptosis of EC cells.

### Silencing AGAP2-AS1 Hinders Tumorigenesis of EC Cells through Downregulating FOSL1 *In Vivo*

**Figure 7:**
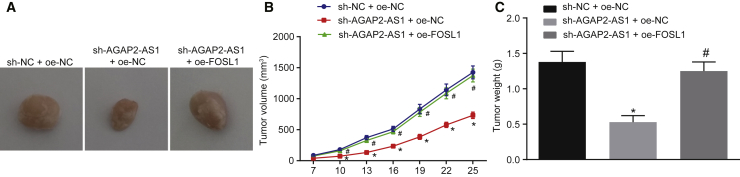
Silencing AGAP2-AS1 Suppresses Tumorigenesis *In Vivo* by Downregulating FOSL1 Nude mice were injected with EC cells that were stably co-transfected with sh-NC and oe-NC, sh-AGAP2-AS1 and oe-NC, or sh-AGAP2-AS1 and oe-FOSL1. (A) Representative images of tumors from nude mice. (B) Tumor volume of nude mice. (C) Tumor weight of nude mice. *p < 0.05 compared with mice that received injection of EC cells stably co-transfected with sh-NC and oe-NC; ^#^p < 0.05 compared with mice that received injection of EC cells stably co-transfected with sh-AGAP2-AS1 and oe-NC. The data are expressed as mean ± standard deviation and were compared by one-way ANOVA, followed by Tukey’s *post hoc* test. Data at different time points were analyzed by repeated-measurement ANOVA, followed by a Bonferroni’s *post hoc* test. n = 8.

Xenograft nude mouse models were established in order to elucidate the role of AGAP2-AS1 in EC *in vivo*. The results revealed that the tumor volume and weight were reduced in mice after silencing AGAP2-AS1. In contrast, the reduced tumor volume and weight induced by AGAP2-AS1 silencing was counteracted following overexpression of FOSL1 (Figures 7A–7C). Hence, silencing of AGAP2-AS1 suppressed *in vivo* tumorigenesis through downregulating FOSL1.

## Discussion

Owing to various factors including ineffective screening tools and advanced disease stage at diagnosis, the 5-year survival rate of EC patients remains disappointingly low.^20^^,^^21^ This highlights the urgent need for novel therapeutic target discovery, in order to enhance the diagnosis and treatment of EC patients. Alteration of lncRNAs has been implicated in multiple malignant tumors, including EC.^22^^,^^23^ Hence, the present study aimed to investigate the role of AGAP2-AS1 in EC. Our findings identified that AGAP2-AS1 binds to miR-195-5p to induce cell proliferation, invasion, and migration and suppress cell cycle arrest as well as apoptosis in EC via upregulation of FOSL1, providing a novel insight into the molecular mechanism by which AGAP2-AS1 influences EC progression.

In the current study, we analyzed the biological roles of AGAP2-AS1 in EC cells. AGAP2-AS1 upregulation was confirmed in EC cells, and suppressed proliferation, invasion, and migration, along with promoted apoptosis, in EC cells were observed following AGAP2-AS1 knockdown. Consistent with the results of our study, accumulating studies have continued to suggest that AGAP2-AS1 is highly expressed in non-small-cell lung cancer and closely correlated with tumor size and advanced pathological stage of non-small-cell lung cancer.^8^^,^^24^ Meanwhile, another study has also implicated the upregulation of AGAP2-AS1 in gastric cancer.^7^ Wang et al.^25^ concluded that the decline of AGAP2-AS1 contributes to enhancement of cell apoptosis, as well as suppression of cell proliferation and invasion together with migration in glioma *in vitro*, which is in line with our findings. In glioblastoma multiforme, cell proliferation, migration, and invasion are also decreased and cell apoptosis is increased following silencing of AGAP2-AS1.^26^

Furthermore, evidence was previously presented that lncRNAs are capable of increasing miRNA-mediated repression of their target mRNAs by interacting with miRNAs as competing endogenous RNA (ceRNA) or miRNA sponges.^27^ The literature has highlighted the critical role of the balance of the lncRNA-miRNA-mRNA regulatory network in many biological processes, and any disturbance of the ceRNA network may lead to different diseases, including cancers.^28^ Our previous study revealed that homeobox D gene cluster antisense growth-associated lncRNA (HAGLR) functions as a regulator of miR-143-5p triggering epithelial-mesenchymal transition and enhancing tumor metastasis in EC.^29^ Our current results further demonstrated that AGAP2-AS1 could bind to miR-195-5p in EC. A previous report clarified the binding relationship between lncRNA XIST and miR-195-5p, which promotes cell proliferation and invasion in osteosarcoma cancer.^30^

In addition, we found the aberrant downregulation of miR-195-5p in EC cells. Also, repression of EC cell proliferation, migration, and invasion along with promoted apoptosis were all triggered by the overexpression of miR-195-5p. Similarly, prior research revealed that the expression of miR-195 is low in ESCC, and that an increase of miR-195 contributes to inhibition of cell proliferation and migration along with invasion via Cdc42.^10^ Another study has also suggested poor expression of miR-195 expression in EC cells, and it highlighted that miR-195 targets HMGA2 to suppress cell proliferation and promoted cell apoptosis.^31^ The repressive role of miR-195-5p in cell proliferation, invasion, and migration has been consistently documented in oral squamous cell carcinoma.^32^ Our data further provided evidence indicating that AGAP2-AS1 exerted a tumor promotive role by binding to miR-195-5p. Through a similar mechanism, lncRNA SNHG1 binds to miR-195-5p to enhance the proliferative and migratory capabilities of hepatocellular carcinoma cells.^33^

miRNAs have been reported to play a crucial role in various biological processes of EC by targeting related mRNAs or genes.^34^ In our study, we identified that FOSL1 was a target gene of miR-195-5p and was negatively regulated by miR-195-5p, which was similarly reported by a prior study that concluded that miR-195-5p directly targets FOSL1 to inhibit cell migration and invasion in prostate cancer.^35^ Moreover, our study also highlighted the high levels of FOSL1 in EC tissue and cells, while the silencing of FOSL1 led to a decrease in EC cell proliferation, invasion, and migration as well as an increase in EC cell apoptosis. FOSL1 (also known as FRA-1) is a member of the FOS family and has been shown to be a crucial factor in cancer cell progression and maintenance of the transformed state.^36^ FOSL1 exerts its function as an oncogene role by dimerizing with proteins in the Jun family to generate the AP-1 complex, which could potentially govern vital cellular processes related to proliferation and apoptosis.^37^ Consistent with our findings, FOSL1 has been reported to be highly expressed in ESCC, and closely related to its poor prognosis.^14^ Additionally, specific upregulation of FOSL1 in triple-negative breast cancer (TNBC) indicated its significant contributions to the progression and metastasis for TNBC.^38^ As suggested *in vitro*, miR-195-5p directly targeted FOSL1 to inhibit EC progression. Moreover, the *in vivo* experiments confirmed that silencing AGAP2-AS1 functioned to suppress tumor growth through inhibition of FOSL1.

### Conclusion

**Figure 8:**
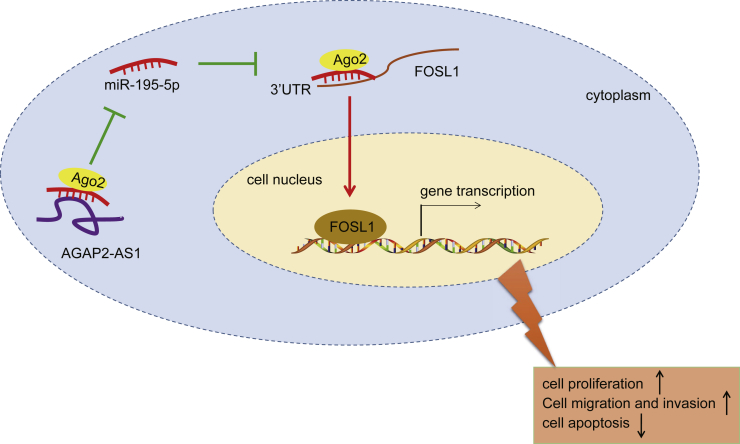
Molecular Mechanisms of AGAP2-AS1/miR-195-5p/FOSL1 Axis on EC AGAP2-AS1 acts as a miR-195-5p sponge to upregulate FOSL1 expression and ultimately promotes proliferation, migration, and invasion, while inhibiting the apoptosis of EC cells.

In conclusion, our results indicated that AGAP2-AS1 was upregulated while miR-195-5p was downregulated in EC. Silencing of AGAP2-AS1 increased miR-195-5p expression to downregulate FOSL1, ultimately suppressing cell proliferation, migration, and invasion while inducing cell cycle arrest and apoptosis in EC (Figure 8). These findings highlight the potential of AGAP2-AS1 as a promising diagnostic biomarker and therapeutic target in EC.

## Materials and Methods

### Ethics Statement

Written informed consent was obtained from all patients prior to the study. Study protocols were approved by the Ethics Committee of the Affiliated Cancer Hospital of Zhengzhou University (Henan Cancer Hospital) following the Declaration of Helsinki. All animal experiments were conducted in strict adherence with the *Guide to the Management and Use of Laboratory Animals* issued by the National Institutes of Health. The animal experiment protocols were conducted with the approval of the Institutional Animal Care and Use Committee of the Affiliated Cancer Hospital of Zhengzhou University (Henan Cancer Hospital).

### Microarray-Based Analysis

EC-related miRNA expression datasets (GEO: GSE43732 and GSE97049) and gene expression datasets (GEO: GSE45670 and GSE45168) were obtained from the GEO database (https://www.ncbi.nlm.nih.gov/geo/). Differential analysis of the normal and tumor samples in EC expression datasets was conducted by the limma package in R language (http://master.bioconductor.org/packages/release/bioc/html/limma.html), and the expression heatmap of differentially expressed miRNAs or DEGs was plotted by the pheatmap package (https://cran.r-project.org/web/packages/pheatmap/index.html). After screening the differentially expressed miRNAs, the target genes of miRNAs were predicted based on five databases, including StarBase (http://starbase.sysu.edu.cn/index.php), DIANA (http://diana.imis.athena-innovation.gr/DianaTools/index.php?r=microT_CDS/index), mirDIP (http://ophid.utoronto.ca/mirDIP/), TargetScan (http://www.targetscan.org/vert_71/), and miRDB (http://www.mirdb.org/), followed by intersecting the target genes with the DEGs screened from GEO: GSE45670. The EC-related genes were investigated using the DisGeNET database (http://www.disgenet.org/web/DisGeNET/menu/search?4). Subsequently, the String database (https://string-db.org/) was adopted for the analysis of the interactions between EC-related genes and DEGs. The PPI network was constructed using Cytoscape 3.6.0 software^39^ to screen the potential genes affecting EC. The relationship between miRNAs and lncRNAs was predicted using the RAID (http://www.rna-society.org/raid/index.html) and StarBase databases. lncRNA subcellular localization was obtained from the LncATLAS database.^40^ The differences between the differentially expressed miRNAs and gene clusters were evaluated using JVenn (http://jvenn.toulouse.inra.fr/app/example.html).

### Clinical Samples

Fifty-three patients with EC (39 males and 14 females, aged 45–74 years, mean age of 57.85 ± 8.06 years) who had received treatment at the Affiliated Cancer Hospital of Zhengzhou University (Henan Cancer Hospital) from May 2017 to October 2018 were enrolled for the study. All patients were confirmed with EC based on histopathological examination. None of the participating patients had received radiation or chemotherapy prior to surgery. Patients who died of other diseases other than EC were excluded from the study. Through surgical excision, tumor and normal adjacent tissues were obtained. The tissue samples were frozen in liquid nitrogen.

### Cell Culture

EC cell lines (KYSE70, KYSE-510, and EC9706) and HEECs were purchased from American Type Culture Collection (Manassas, VA, USA). The cells were cultured Roswell with Park Memorial Institute1640 complete medium (Gibco-BRL, Gaithersburg, MD, USA) containing 10% fetal bovine serum (FBS, Gibco-BRL, Gaithersburg, MD, USA) at 37°C with 5% CO_2_. The complete medium was renewed at regular 2- to 3-day intervals, and the cells were detached and passaged conventionally once the confluence reached 80%.

### Cell Treatment and Transfection

The EC cells were transfected with the following sequences or plasmids: shRNA against AGAP2-AS1 (sh-AGAP2-AS1), miR-195-5p mimic, miR-195-5p inhibitor, and FOSL1 overexpression plasmid (oe-FOSL1) as well as corresponding negative controls (sh-NC, oe-NC, NC-mimic, and NC-inhibitor). The cells at the logarithmic phase were prepared into a single-cell suspension that was subsequently seeded into a six-well plate. After cell confluence had reached 80%, transfection was performed in accordance with the instructions of the Lipofectamine 2000 kit (11668-019, Invitrogen, Carlsbad, CA, USA). After a 48-h period of transfection, the cells were harvested for the subsequent experiments. All sequences and plasmids were purchased from Shanghai GenePharma (Shanghai, China).

### RNA isolation and quantitation

Following isolation of total RNA from the cells with TRIzol reagent (Invitrogen, Carlsbad, CA, USA), the concentration and purity of the total extracted RNA were determined using a NanoDrop 2000 spectrophotometer (1011U, NanoDrop Technologies, Wilmington, DE, USA). A TaqMan miRNA reverse transcription kit (4427975, Applied Biosystems, Branchburg, NJ, USA) and a PrimeScript RT reagent kit (RR047A, Takara, Kyoto, Japan) were applied for reverse transcription. An ABI 7500 quantitative real-time PCR system (Applied Biosystems, Foster City, CA, USA) was employed to conduct quantitative real-time PCR reactions. The expression of miR-195-5p was normalized to the housekeeping gene U6, while the expression of other genes was normalized to housekeeping gene glyceraldehyde-3-phosphate dehydrogenase (GAPDH). The gene expression fold changes were calculated based on the 2^−ΔΔCt^ method.^41^ The primers for AGAP2-AS1 and miR-195-5p were designed and synthesized by Takara Biotechnology (Dalian, Liaoning, China) (Table 1).

**Table 1:** Primer Sequences for qRT-PCR

Genes	Primer Sequences
AGAP2-AS1	F: 5′-TACCTTGACCTTGCTGCTCTC-3′
	R: 5′-TGTCCCTTAATGACCCCATCC-3′
miR-195-5p	F: 5′-ACACTCCAGCTGGGTAGCAGCACAGAAAT-3′
	R: 5′-TGGTGTCGTGGAGTCG-3′
U6	F: 5′-CTCGCTTCGGCAGCACA-3′
	R: 5′-AACGCTTCACGAATTTGCGT-3′
GAPDH	F: 5′-TCAAGGCTGAGAACGGGAAG-3′
	R: 5′-TGGACTCCACGACGTACTCA-3′

F, forward; R, reverse; qRT-qPCR, quantitative reverse transcription polymerase chain reaction; GAPDH, glyceraldehyde-3-phosphate dehydrogenase.

### Western Blot Analysis

Radioimmunoprecipitation assay lysis buffer containing phenylmethanesulfonylfluoride was utilized for isolation of total protein of tissues or cells, followed by a 30-min period of incubation on ice and a 10-min centrifugation at 8,000 × *g* at 4°C. The protein concentration of the collected supernatant was determined using a bicinchoninic acid protein quantification kit. Following separation of the protein via sodium dodecyl sulfate-polyacrylamide gel electrophoresis, the protein was transferred onto a polyvinylidene fluoride membrane. The membrane was then blocked using 5% skimmed milk at 37°C for 1 h, followed by membrane incubation at 4°C overnight with the primary rabbit polyclonal antibodies to FOSL1 (ab232745, 1:1,000) or GAPDH (ab9485, 1:2,500). The membrane was rinsed three times with Tris-buffered saline with Tween 20 (TBST; 10 min each time), followed by a 1-h incubation with the secondary goat anti-rabbit IgG antibody tagged by horseradish peroxidase (ab97051, 1:2,000). All antibodies were purchased from Abcam (Cambridge, UK). Following development with an enhanced chemiluminescence fluorescence detection kit (BB-3501, Amersham Pharmacia, Piscataway, NJ, USA), images were captured using the Bio-Rad image analysis system (Bio-Rad, Hercules, CA, USA). The relative expression of the proteins was analyzed using Quantity One v4.6.2 software and expressed as the ratio of gray value of the corresponding protein band to that of the GAPDH band.

### Fractionation of Nuclear and Cytoplasmic RNA

The nuclear and cytoplasmic RNA fractions were isolated using a PARIS kit protein and RNA isolation system (Life Technologies, Carlsbad, CA, USA). After trypsinization, a 5-min centrifugation was performed at 500 × *g*. The cells were subsequently triturated by utilizing 500 μL of cell fractionation buffer, together with incubation on ice for 5–10 min. Next, the supernatant was obtained using a 5-min centrifugation at 500 × *g* and 4°C. The supernatant (cytoplasmic fraction) was centrifuged in a new 2-mL sterile enzyme-free tube at 500 × *g* and 4°C for 5 min. After the precipitation (nuclear fraction) was resuspended in 500 μL of cell fractionation buffer, the nuclear fraction was separately rinsed in 500 μL of 2× lysis/binding solution. After centrifugation, the pellet was resuspended in pre-cooled 500 μL of cell fractionation buffer and 500 μL of absolute ethanol and transferred into the adsorption column, which was subsequently placed into a collection tube. After centrifugation and washing, the nucleus RNA was harvested by elution. AGAP2-AS1 expression was assessed by performing qRT-PCR, with 45S rRNA as the internal reference for nuclear RNA and 12S rRNA for cytoplasmic RNA. The primers are depicted in Table 2.

**Table 2:** Primer Sequences for qRT-PCR

Target Genes	Primer Sequences
45S rRNA	F: 5′-GTGCCCTCACGTGTTTCACTTT-3′
	R: 5′-TAGGAGACAAACCTGGAACGCT-3′
12S rRNA	F: 5′-TCGATAAACCCCGCTCTACCT-3′
	R: 5′-TGGCTACACCTTGACCTAACGTT-3′

F, forward; R, reverse; qRT-PCR, quantitative reverse transcription polymerase chain reaction.

### Dual-Luciferase Reporter Assay

FOSL1-3′UTR fragments and AGAP2-AS1 cDNA fragments that containing the miR-195-5p binding site were inserted into the pGL3 plasmids to construct a luciferase reporter vector. The FOSL1-3′ UTR-MUT fragment and the AGAP2-AS1-MUT fragment with the miR-195-5p mutated binding site were also inserted into the pGL3 vector. Using the liposome transfection method, recombinant plasmids (pGL3-AGAP2-AS1, pGL3-AGAP2-AS1-MUT, pGL3-FOSL1-3′ UTR, pGL3-FOSL1-3′ UTR-MUT) and Renilla reference plasmid were respectively co-transfected with miR-195-5p mimic or NC-mimic into HEK293T cells. Forty-eight hours after transfection, luciferase activity was determined using a luciferase reporter assay system (Promega, Madison, WI, USA), together with the utilization of a luciferase assay kit (K801-200, BioVision Technologies, San Francisco, CA, USA). With Renilla luciferase as the internal reference, activation of the target reporter gene was considered to be reflective of the ratio of the relative luciferase unit value of firefly luciferase to that of Renilla luciferase.

### 5-Ethynyl-2′-Deoxyuridine (EdU) Assay

EC cells at the logarithmic phase were seeded into a 96-well plate (4 × 10^4^ cells/well). After treatment for 24 h, 100 μL of EdU medium was added to the cells in each well for a 2-h incubation, followed by fixation for 30 min with 100 μL of cell fixative at room temperature. Cells in each well were incubated with 2 mg/mL glycine for 5 min and with 100 μL of phosphate-buffered saline (PBS) containing 0.5% Triton X-100 for 10 min. Next, the cells in each well were stained with 100 μL of Apollo 567 (Guangzhou Ribobio Biotechnology, Guangzhou, Guangdong, China) for 30 min and 100 μL of 1× Hoechst 33342 reaction solution. After sealing in 100 μL of anti-fluorescence quenching agent, the cells were imaged under a fluorescence microscope, with the number of cells labeled with EdU counted. The EdU labeling rate (%) = [the number of EdU-positive cells/(the number of EdU-positive cells + EdU-negative cells)] × 100%.

### Transwell Assay

Matrigel (YB356234, Shanghai Yubo Biological Technology, Shanghai, China) was thawed overnight at 4°C, and 200 μL of serum-free medium was employed for dilution at 4°C. A total of 50 μL of diluted Matrigel was added to the apical chamber of the Transwell plate. The cells were trypsinized, counted, and resuspended into a single-cell suspension. The cell suspension (200 μL) was added into the apical chamber of each well. A total amount of 800 μL of conditioned culture medium containing 20% FBS was then added into the basolateral chamber. The cell incubation was conducted at 37°C for 20–24 h. The cells were then rinsed twice with PBS, fixed with formaldehyde for 10 min, washed three times with water, and stained with 0.1% crystal violet for 30 min at room temperature. The numbers of cells remaining in the basolateral chamber were observed, imaged, and counted under an inverted microscope. Using a Transwell migration assay, the cells were incubated for 16 h without Matrigel. In order to assess the invasion and migration abilities, the number of cells invaded through the Matrigel and migrated into the basolateral chamber in four random visual fields was then counted.

### Flow Cytometry

Following a 48-h transfection, the cells were trypsinized and dispersed into cell suspension (1 × 10^6^ cells/mL). Next, 1 mL of cell suspension was centrifuged at 1,500 rpm for 10 min. The pellet was added with 2 mL of PBS and centrifuged again. The harvested pellet was fixed with 70% ethanol at 4°C overnight. Next, 100 μL of cell suspension was stained with 50 μg of 1% propidium iodide (PI) containing RNAase for 30 min. The cell cycle distribution was then detected on a flow cytometer (BD Biosciences, Franklin Lakes, NJ, USA).

Cell apoptosis was assessed using annexin V-fluorescein isothiocyanate (FITC)/PI double staining. The cells were incubated in an incubator at 37°C with 5% CO_2_ for 48 h. The harvested cells were rinsed with PBS and then resuspended in 200 μL of binding buffer. The cell suspension was then permitted to react with the 10 μL of annexin V-FITC (ab14085, Abcam, Cambridge, UK) and 5 μL of PI at room temperature under conditions void of light for 15 min at room temperature. The FITC and PI signals were detected using a flow cytometer at an excitation wavelength of 488 nm.

### RNA Pull-Down

The EC cells were subsequently treated with 50 nM biotinylated Bio-miR-195-5p-probe and Bio-NC-probe (Genecreate Bioengineering, Wuhan, Hubei, China). After 48 h, the cells were incubated for 10 min in Pierce IP lysis buffer (Thermo Fisher Scientific, Rockford, IL, USA). Streptavidin magnetic beads (Thermo Fisher Scientific, Rockford, IL, USA) were then added to the lysate for incubation at 4°C overnight. The cells were then washed twice with pre-chilled lysis buffer, three times with low-salt buffer, and once with high-salt buffer. After collection of the supernatant by magnetic separation, the bound RNA was purified by TRIzol, with AGAP2-AS1 enrichment detected by qRT-PCR.

### RIP Assay

After lysing in an ice bath with an equal volume of RIP lysis buffer (P0013B, Beyotime Biotechnology, Shanghai, China) for 5 min, the cells were centrifuged for 10 min at 14,000 rpm and 4°C. According to the protocol of the RIP assay kit (Millipore, Billerica, MA, USA), the binding between AGAP2-AS1 and the protein Ago2 was assessed. Next, 100 μL of RIP wash buffer was added to resuspend 50 μL of magnetic beads in each coprecipitation reaction system, which was then cultured at 4°C with 5 μg of rabbit antibody to Ago2 (ab186733, 1:50, Abcam, Cambridge, UK) for more than 6 h. Following the addition of 900 μL of RIP wash buffer, the magnetic bead-antibody complex was resuspended and incubated overnight at 4°C with 100 μL of cell supernatant. The magnetic bead-protein complex was obtained by placing the sample onto a magnetic stand. The sample and input were separately trypsinized with proteinase K, with the RNA extracted for subsequent quantitative real-time PCR detection. The rabbit antibody to IgG (ab172730, 1:100, Abcam, Cambridge, UK) was regarded as the NC.

### Xenograft Tumor in Nude Mice

A total of 24 specific pathogen-free female BALB/c nude mice (aged 6 weeks, weighing 15–18 g) were purchased from Shanghai SLAC Laboratory Animal Co. (Shanghai, China). The KYSE70 cells were stably co-transfected with sh-NC and oe-NC, sh-AGAP2-AS1 and oe-NC, or sh-AGAP2-AS1 and oe-FOSL1. The animals were divided into three groups (eight mice in each group) and injected subcutaneously with the stably transfected KYSE70 cells (2 × 10^6^ cells/mL). After 4 weeks, the BALB/c nude mice were euthanized, and their tumors were collected and weighed.

### Statistical Analysis

All statistical analyses were conducted using SPSS 21.0 statistical software (IBM, Armonk, NY, USA). Measurement data were expressed as mean ± standard deviation. The differences between the normally distributed values of two experimental groups were analyzed by a paired t test or unpaired t test. Data among multiple groups were analyzed by one-way analysis of variance (ANOVA), followed by Tukey’s *post hoc* test. Statistical analysis in relationship to time-based measurements within each group was conducted using repeated-measures ANOVA, followed by Bonferroni’s *post hoc* test. p < 0.05 was considered to be indicative of a statistically significant difference.

## Author Contributions

S.S., K.L., Y.L., and X.L. designed the study. S.S., B.L., and W.X. collated the data, carried out data analyses, and produced the initial draft of the manuscript. Y.B., W.X., and S.S. contributed to drafting the manuscript. All authors have read and approved the final submitted manuscript.

## Conflicts of Interest

The authors declare no competing interests.
